# Mitochondrial angiotensin receptors in dopaminergic neurons. Role in cell protection and aging-related vulnerability to neurodegeneration

**DOI:** 10.1038/cddis.2016.327

**Published:** 2016-10-20

**Authors:** Rita Valenzuela, Maria A Costa-Besada, Javier Iglesias-Gonzalez, Emma Perez-Costas, Begoña Villar-Cheda, Pablo Garrido-Gil, Miguel Melendez-Ferro, Ramon Soto-Otero, Jose L Lanciego, Daniel Henrion, Rafael Franco, Jose L Labandeira-Garcia

**Affiliations:** 1 Laboratory of Neuroanatomy and Experimental Neurology, Department of Morphological Sciences, CIMUS, University of Santiago de Compostela, Santiago de Compostela, Spain; 2 Networking Research Center on Neurodegenerative Diseases (CIBERNED), Madrid, Spain; 3 Healing Foundation Centre, The University of Manchester, Manchester, UK; 4 Department of Pediatrics-Pediatric Nephrology, University of Alabama at Birmingham, Birmingham, AL, USA; 5 Department of Surgery-Pediatric, University of Alabama at Birmingham, Birmingham, AL, USA; 6 Laboratory of Neurochemistry, Department of Biochemistry and Molecular Biology, Faculty of Medicine, University of Santiago de Compostela, Santiago de Compostela, Spain; 7 Neuroscience Department, Center for Applied Medical Research (CIMA, IdiSNA), University of Navarra, Pamplona, Spain; 8 MITOVASC Institute, INSERM U1083, CNRS UMR6214, University of Angers, Angers, France; 9 Laboratory of Molecular Neurobiology, Department of Biochemistry and Molecular Biology, Faculty of Biology, University of Barcelona, Barcelona, Spain

## Abstract

The renin–angiotensin system (RAS) was initially considered as a circulating humoral system controlling blood pressure, being kidney the key control organ. In addition to the ‘classical' humoral RAS, a second level in RAS, local or tissular RAS, has been identified in a variety of tissues, in which local RAS play a key role in degenerative and aging-related diseases. The local brain RAS plays a major role in brain function and neurodegeneration. It is normally assumed that the effects are mediated by the cell-surface-specific G-protein-coupled angiotensin type 1 and 2 receptors (AT1 and AT2). A combination of *in vivo (rats, wild-type mice and* knockout *mice)* and *in vitro* (primary mesencephalic cultures, dopaminergic neuron cell line cultures) experimental approaches (confocal microscopy, electron microscopy, laser capture microdissection, transfection of fluorescent-tagged receptors, treatments with fluorescent angiotensin, western blot, polymerase chain reaction, HPLC, mitochondrial respirometry and other functional assays) were used in the present study. We report the discovery of AT1 and AT2 receptors in brain mitochondria, particularly mitochondria of dopaminergic neurons. Activation of AT1 receptors in mitochondria regulates superoxide production, via Nox4, and increases respiration. Mitochondrial AT2 receptors are much more abundant and increase after treatment of cells with oxidative stress inducers, and produce, via nitric oxide, a decrease in mitochondrial respiration. Mitochondria from the nigral region of aged rats displayed altered expression of AT1 and AT2 receptors. AT2-mediated regulation of mitochondrial respiration represents an unrecognized primary line of defence against oxidative stress, which may be particularly important in neurons with increased levels of oxidative stress such as dopaminergic neurons. Altered expression of AT1 and AT2 receptors with aging may induce mitochondrial dysfunction, the main risk factor for neurodegeneration.

The renin–angiotensin system (RAS) was initially considered as a circulating humoral system controlling blood pressure and kidney as a key control organ. The actions of angiotensin II (AII), the most important effector peptide, are mediated by two main cell receptors: AII type 1 and 2 (AT1 and AT2). It is generally considered that AT2 receptors exert actions directly opposed to those mediated by AT1 receptors, thus antagonizing many of the effects of the latter.^1^ In addition to this ‘classical' humoral RAS, a second RAS, local or tissue RAS, has been identified in a variety of tissues, including the central nervous system.^2^ The role of RAS on brain function was initially associated with the effects of circulating RAS in areas involved in central control of blood pressure; however, it is now known that the local brain RAS is involved in different brain functions and disorders.^3, 4^ We have previously demonstrated the presence of a local RAS in the substantia nigra pars compacta (SNc) and striatum of rodents and primates, including humans.^5, 6, 7^ It has also been demonstrated that overactivation of local RAS, via AT1 receptors, exacerbates neuroinflammation, oxidative stress and dopaminergic cell death, all of which are inhibited by treatment with AT1 receptor antagonists.^8, 9^

More recently, a third-level of RAS (i.e. intracellular/intracrine) has been suggested in peripheral tissues.^10, 11^ The system may be activated by AII internalized using AT1 receptors or by intracellularly synthesized AII.^12^ Immunohistochemical studies suggest an apparent intracellular localization of several RAS components in the SNc of mammals, including primates and humans.^5, 13^ Mitochondrial dysfunction plays a major role in several neurodegenerative disorders, particularly in the degeneration of dopaminergic neurons in Parkinson's disease (PD). In the present study, we have discovered AT1 and AT2 receptors in brain mitochondria and investigated their role in controlling mitochondrial events. The experiments were carried out in rats, in AT1 and AT2 receptor knockout mice, in primary cultures of the nigral region and in the dopaminergic neuron cell line MES 23.5. We carried out functional studies with isolated mitochondria to exclude any possible indirect effects caused by non-mitochondrial AT1 and AT2 receptors, and showed that angiotensin receptors control key mitochondrial events.

## Results

### Localization of angiotensin receptors in mitochondria of dopaminergic neurons in cell cultures and rat substantia nigra

**Figure 1:**
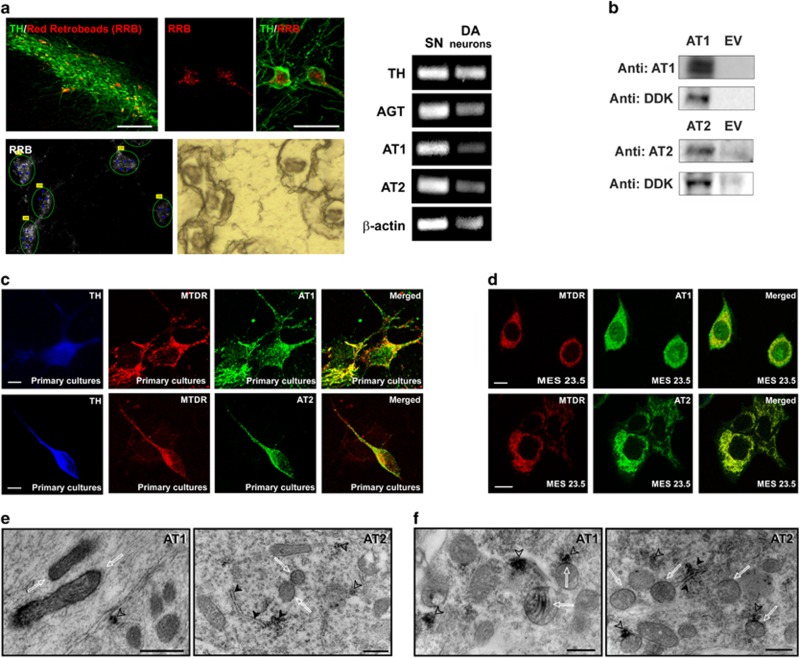
Specificity of angiotensin receptor antibodies and localization of angiotensin receptors in mitochondria of dopaminergic neurons. (**a**) The expression of major RAS components in dopaminergic neurons was confirmed by RT-PCR and laser microdissection of dopaminergic neurons retrogradly labeled by intrastriatal injection of fluorescent red retrobeads (RRB). SN section showing labeled dopaminergic neurons before and after laser microdissection for RT-PCR. Expression of TH, AGT, AT1, AT2 and *β*-actin mRNA in laser-microdissected dopaminergic neurons (right) and homogenates of SN used as a positive control (left) are also shown. (**b**) Western blot densitometric bands corresponding to 10 *μ*g of AT1 or AT2 receptor overexpression lysate containing a C-terminal DDK epitope tag (DYKDDDDK) fused (left), and 10 *μ*g of empty vector transfected control cell lysate HEK293 (EV, right). A band of 45 kDa was detected with the AT1 antibody, while a band of 50 kDa was detected with the AT2 antibody. A monoclonal antibody against DDK detected the corresponding band in the protein lysates. Colocalization of mitochondria and AT1 and AT2 receptors in primary cultures of the nigral region (**c**) and neurons from the MES 23.5 dopaminergic cell line (**d**). Electron microscopy of AT1 and AT2 labeling in a neuronal process (**e**) and cytoplasm (**f**) of a dopaminergic neuron. Immunolabeling for AT1 was observed in the outer membrane and cristae (white arrows) of mitochondria. In addition, strong AT1 labeling was also seen in clusters of free ribosomes (empty arrowheads). Immunolabeling for AT2 was present in mitochondrial membranes (white arrows), in the rough endoplasmic reticulum (black arrowheads) and in small clusters of free ribosomes, some of which were in close proximity to labeled mitochondria (empty arrowheads). AGT, angiotensinogen; DA, dopaminergic; MTDR, MitoTracker Deep Red; SN, substantia nigra; TH, tyrosine hydroxylase. Scale bars: (**a**) 50 and 200 *μ*m (SN section); (**c**) 5 *μ*m; (**d**) 10 *μ*m; (**e** and **f**) 500 nm

The localization of AT1 and AT2 receptors in dopaminergic neurons has been shown by immunohistochemistry in previous studies. In the present study, this was confirmed by laser captured microdissection (LCM) of dopaminergic neurons in the rat substantia nigra (SN) and reverse trascription polymerase chain reaction (RT-PCR). RT-PCR analysis revealed expression of detectable mRNA levels of TH, angiotensinogen and AT1 and AT2 receptors in isolated nigral dopaminergic neurons (Figure 1a).

We used triple immunofluorescence and confocal microscopy to investigate whether mitochondria of dopaminergic neurons expressed AT1 and AT2 receptors. Series of confocal images were obtained every 0.7 *μ*m in the *Z*-axis level by a sequential scan method. Dopaminergic neurons were identified by their tyrosine hydroxylase (TH)-immunoreactivity, and mitochondria were labeled with the specific probe Mitotracker Deep Red (MTDR), which revealed the characteristic pattern of the mitochondrial network. The specificity of the antibodies (see Materials and Methods) was confirmed by western blot (WB) analysis of lysates from HEK293 cells transfected with recombinant AT1 or AT2 containing a C-terminal DDK epitope tag (DYKDDDDK) fused (Origene). A predominant immunoreactive band was observed in positive transfected lysates but not in negative controls, which consisted of empty vector transfected lysates (Figure 1b).

In primary cultures (Figure 1c) and the MES 23.5 cell line of dopaminergic (i.e. TH-positive) neurons (Figure 1d), dopaminergic neurons showed intense inmunoreactivity to TH antibody. Labeling for AT1 receptors was intense at the periphery of the cells, suggesting the presence of receptors at the cell surface, and also within the neurons, at cytoplasmic and nuclear levels. Colocalization of AT1 with the specific probe MTDR revealed that many of the mitochondria expressed AT1 receptors, although some mitochondria showed weak or unclear AT1 immunolabeling. On the contrary, AT2 receptor labeling was particularly intense in the cytoplasm, while weaker labeling was observed at the cell surface and in the nucleus. At the cytoplasmic level, AT2 labeling colocalized with MTDR and the pattern of distribution was similar to that of mitochondrial labeling, which suggests a strong presence of this receptor in mitochondria of dopaminergic neurons.

Electron microscopy samples were obtained from the densely packed dopaminergic cell clusters of the rat substantia nigra compacta. Labeling for both AT1 and AT2 receptors was observed in the neuronal processes (Figure 1e) and in the cytoplasm of dopaminergic neurons (Figure 1f), associated with different organelles and cytoplasmic structures. More specifically, labeling was frequently observed in mitochondria, mainly located in the outer membrane (Figures 1e and f) although in some cases labeling was also observed in the cristae (Figure 1f, left). Labeling for both receptors was also observed in the rough endoplasmic reticulum, and in clusters of free ribosomes, which were often located in close vicinity to labeled mitochondria (Figures 1e and f).

### Presence of fluorescent-tagged angiotensin receptors and fluorescent AII in mitochondria

**Figure 2:**
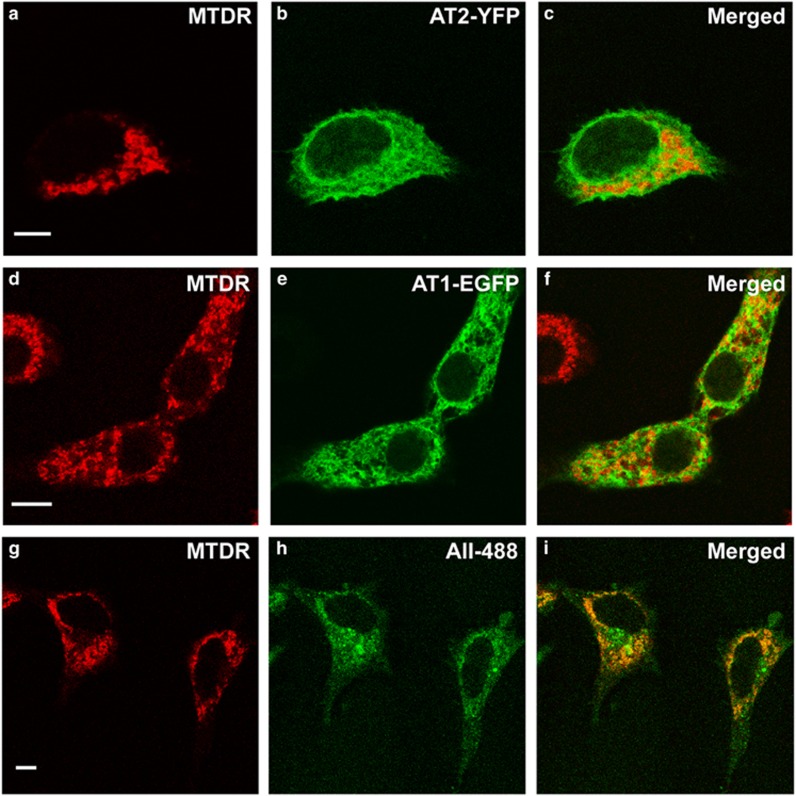
Presence of fluorescence-tagged angiotensin receptors and fluorescent angiotensin II in mitochondria. Colocalization (**c**, **f** and **i**) of the fluorescent mitochondrial marker MTDR (**a**, **d** and **g**) with AT2-YFP (**b**), AT1-EGFP (**e**) or AII-488 (**h**). Fluorescent AII-488 colocalized with mitochondria 8 h after treatment of cultures (**g–i**). Cells transfected with AT1-EGFP required simultaneous treatment with the AT1 receptor antagonist losartan, in order to minimize AT1-induced superoxide toxicity and cell death. AT2-YFP, angiotensin receptor type 2 tagged to yellow fluorescent protein; AT1-EGFP, angiotensin receptor type 1 tagged to enhanced green fluorescent protein; AII-488, Alexa Fluor 488-fluorescent angiotensin II; MTDR, MitoTracker Deep Red. Scale bar: 5 *μ*m

Twenty-four hours after transient transfection of the dopaminergic neuron cell line MES 23.5, AT1 and AT2 receptors labeled with fluorescent proteins (yellow fluorescent protein, YFP; enhanced green fluorescent protein, EGFP) were located both at the plasma membrane and intracellularly, and they colocalized with the mitochondrial marker MDTR (Figures 2a–f). In a second series of experiments, cultures of the dopaminergic neuron cell line MES 23.5 were treated with Alexa Fluor 488-conjugated AII to investigate the localization of AII within the mitochondria. The fluorescent AII was internalized and colocalized with mitochondria, which was evident after 30 min, and persisted for 8 h after treatment (Figures 2g–i).

Interestingly, enhanced intracellular oxidative stress (i.e. after treatment of cells with very low doses of MPP^+^) altered the distribution of AT2-YFP labeling. In particular, after labeling mitochondria with a mitotracker probe, AT2-YFP was more abundant at the mitochondrial level than in untreated cells. However, treatment of cells with the antioxidant *N*-acetyl-cysteine (NAC) induced increased mitochondrial AT1-EGFP fluorescence relative to control cells. These effects were confirmed by WB in samples from isolated mitochondria (see below).

### Presence of angiotensin receptors and AII in isolated mitochondria

**Figure 3:**
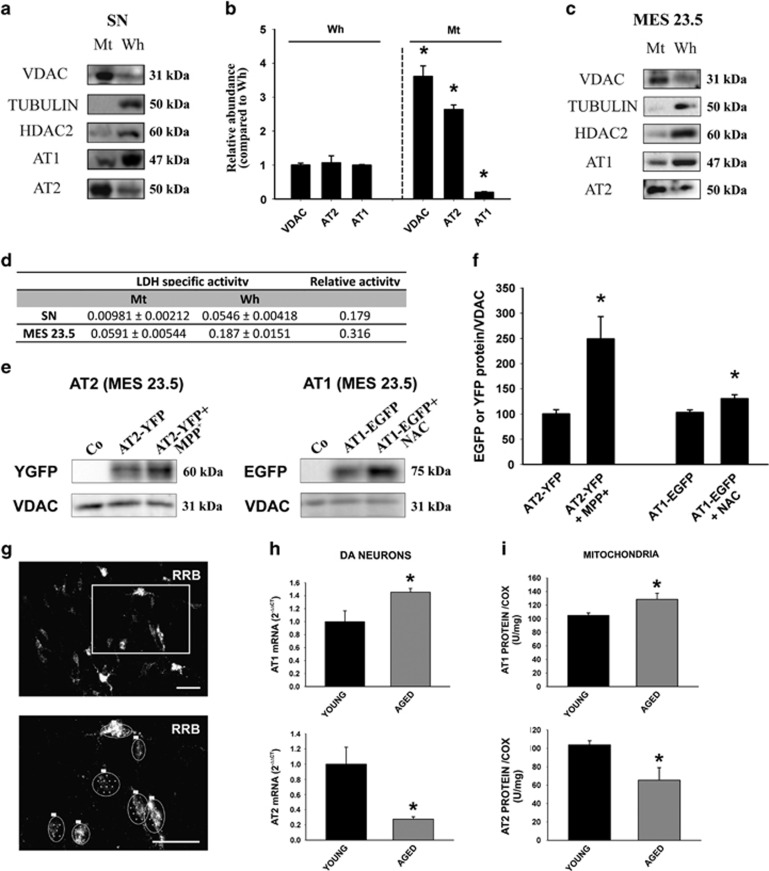
AT1 and AT2 receptors in isolated mitochondria (IM) from ventral mesencephalon and the dopaminergic neuron cell line MES 23.5. Effect of oxidative stress and aging. (**a–c**) Western blot (WB) of whole homogenate (Wh; *n*=4) and pure IM (Mt; *n*=4) from the nigral region (**a**) and MES 23.5 cells (**c**) showing different compartment markers used to assess the purity of the IM: voltage-dependent anion channel (VDAC) as a mitochondrial marker, tubulin as a cytosol marker and histone deacetylase 2 (HDAC2) as a nuclear marker. Note the higher expression of mitochondrial AT2 compared with AT1 receptors (**b**). (**d**)The levels of lactate dehydrogenase specific activity (LDH; micromol of substrate/min/mg; a marker for cytosolic and synaptosomal contamination) of the nigral region and MES 23.5 pure IM (Mt; *n*=6) were negligible relative to those in Wh. (**e** and **f**) WB of IM from the MES 23.5 cells transfected with AT2-YFP (yellow fluorescent protein; *n*=4) or AT1-EGFP (enhanced green fluorescent protein; *n*=3) showing the presence of fluorescent-tagged angiotensin receptors in IM in comparison with non-transfected cells (Co, control); 24 h treatment of cells, low doses of MPP^+^ (*n*=4) or *N*-acetyl-l-cysteine (NAC; *n*=3) induced increased expression of mitochondrial AT2 and AT1 receptors, respectively (AT2-YFP+MPP^+^; AT1-EGFP+NAC). (**g–i**) In young (*n*=3) and aged (*n*=4) rats, the expression of AT1 and AT2 receptors was analyzed by RT-PCR in nigral dopaminergic neurons labeled with red retrobeads (RRB) and isolated by laser microdissection (**g–h**), and by WB of IM from the nigral region of young (*n*=5–8) and aged rats (*n*=5-6) (**i**). Aging induced a significant increase in AT1 expression and a significant decrease in AT2 expression in both dopaminergic neurons and in IM. The results were normalized to the values of young animals. Data are means±S.E.M. *P<0.05 relative to the corresponding controls (Student's *t*-test). Scale bars: 50 *μ*m

Mitochondria were isolated from the nigral region of rat in the ventral mesencephalon (VM) by separating organelles by ultracentrifugation in a preformed Percoll gradient.^14^ The quality of the sample was demonstrated by the absence of different specific cell compartment markers, such as histone deacetylase 2 (HDAC2) and *α*-tubulin, which are markers for the nuclear and the cytosol fractions, respectively. The mitochondrial fraction was confirmed with the mitochondrial marker voltage-dependent anion channel (VDAC). The activity of lactate dehydrogenase (LDH), which is a cytosolic enzyme predominantly associated with contaminating synaptosomes, was measured relative to that of the whole homogenate, and only residual nonsignificant activity was observed in the mitochondrial fraction (Figures 3a–d). We observed the presence of the main angiotensin receptor types (i.e. AT1 and AT2) in the mitochondrial fraction. There was a clear difference in the abundance of each receptor subtype; while AT1 appeared less abundant in mitochondria than in total cell homogenate, AT2 expression was higher than in the total homogenate (Figures 3a–c). Furthermore, we have confirmed the presence of AII peptide in isolated mitochondria using HPLC purified samples (0.0381±0.00618 pg/ml).

### Effects of oxidative stress levels and aging on the expression of mitochondrial angiotensin receptors

After transient transfection of the dopaminergic neuron cell line MES 23.5, AT2-YFP was more abundant at the mitochondrial level after treatment of cells with inducers of oxidative stress such as very low doses of MPP^+^; relative density of the protein bands normalized to VDAC were 94.9±13.0 for untreated controls and 248.8±44.3 for cells treated with MPP^+^ (*P*<0.05). Treatment of cells with the antioxidant NAC led to higher mitochondrial levels of AT1-EGFP than in mitochondria from untreated dopaminergic cells (Control: 103.1±5.0; NAC: 130.6±7.8; *P*<0.05; Figures 3e and f).

RT-PCR analysis of dopaminergic neurons isolated from rat SN using LCM revealed that aging induces a significant increase (45%) in AT1 receptor mRNA expression. In contrast, the levels of AT2 receptor mRNA in dopaminergic neurons were significantly lower (83% decrease) in aged rats than in young rats (Figures 3g and h). In isolated mitochondria, the expression of mitochondrial AT1 receptors detected by WB increased in aged rats, while expression of mitochondrial AT2 was lower in aged rats than in young rats (Figure 3i). Furthermore, using the same isolation procedure, there was a substantial difference in total protein concentration between mitochondria isolated from young and aged rats. Mitochondria isolated from aged rats contained almost half the amount of protein concentration than those from young rats. This is not surprising since the number and mass of mitochondria is known to decrease with age, which alters the quantification of protein between both groups of animals. Therefore, to obtain a more accurate estimation of mitochondrial content, we used cytochrome *c* oxidase (COX) activity to normalize the data obtained from AT1 and AT2 receptor expression determined by WB (Figure 3i).^15^

### Effect of mitochondrial angiotensin receptors on mitochondrial respiration

**Figure 4:**
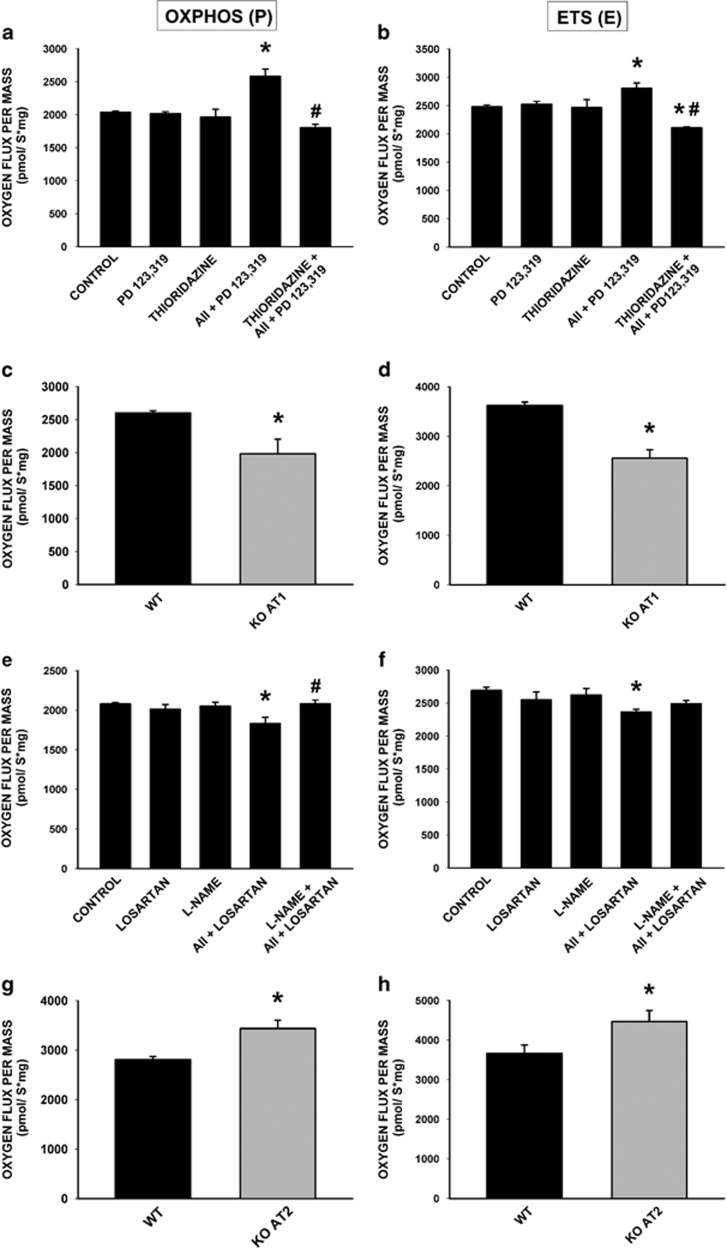
Effect of mitochondrial angiotensin receptors on mitochondrial respiration. (**a** and **b**) Activation of mitochondrial AT1 receptors with AII (i.e. AII+ PD123,319) induces an increase in both oxidative phosphorylation (P) and maximum respiratory rate (E), which was inhibited by pre-treatment of isolated mitochondria with the NOX4 inhibitor thioridazine (*n*=3–8). (**c** and **d**) Knockout mice for AT1 receptors (KO AT1; *n*=5) show lower mitochondrial respiration rates compared with wild-type littermate controls (WT). (**e** and **f**) Activation of mitochondrial AT2 receptors by AII (i.e. AII+losartan) produces a significant decrease in activated respiration (OXPHOS, P) and maximum respiration rate (maximum electron transport system, ETS, E) associated with complex I, which was blocked by pre-incubation of isolated mitochondria with the nitric oxide synthase (NOS) inhibitor, *N^ω^*-nitro-l-arginine methyl ester hydrochloride (l-NAME; *n*=5–8). (**g** and **h**) Mice lacking AT2 receptors (KO AT2; *n*=5) show an increased respiratory activity compared with wild-type mice (WT). Data are mean±S.E.M. *P<0.05 compared with control. ^#^*P*<0.05 compared with the group treated with AII. One-way analysis of variance (ANOVA) and Bonferroni *post hoc* test (**a**, **b**, **e**, **f**) and Student's *t*-test(**c**, **d**, **g**, **h**)

Bioenergetic studies were carried out using mitochondria isolated from the nigral region of rat, and confirmed with mitochondria isolated from the whole brain. Isolated mitochondria were treated with AII in the presence of antagonists of either AT1 (losartan) or AT2 (PD123,319) receptors. Our results showed that activation of mitochondrial angiotensin receptors produced opposite effects on respiratory function. Activation of mitochondrial AT1 receptors with AII (i.e. AII+ PD123,319) induced an increase in both oxidative phosphorylation (P) and maximum respiratory rate (E) (Figures 4a and b). This was consistent with that observed in mice lacking AT1 receptors, which showed a decrease in respiratory activity compared with wild-type mice (Figures 4c and d). Activation of mitochondrial AT2 receptors by AII (i.e. AII+losartan) produced a significant decrease in activated respiration (OXPHOS or P) and maximum respiration rate (maximum electron transport system, ETS or E) associated with complex I (Figures 4e and f). This is consistent with the results in knockout mice for AT2 receptors, which showed higher mitochondrial respiration rates compared with wild-type littermate controls (Figures 4g and h).

Interestingly, the increase in respiratory capacity induced by activation of mitochondrial AT1 receptor was blocked by pre-incubation of isolated mitochondria with the NOX4 inhibitor thioridazine, which revealed the major role of NOX4 in the induction of superoxide by mitochondrial AT1 receptor activation (Figures 4a and b). The decrease in respiratory capacity induced by activation of mitochondrial AT2 receptors was inhibited by pre-treatment of isolated mitochondria with the nitric oxide synthase (NOS) inhibitor, *N^ω^*-nitro-l-arginine methyl ester hydrochloride (l-NAME), which revealed the major role of nitric oxide (NO) in this effect (Figures 4e and f).

### Effect of mitochondrial angiotensin receptors on mitochondrial transmembrane potential, nitric oxide and superoxide production. Role of mitochondrial NOS and mitochondrial NADPH oxidase 4

**Figure 5:**
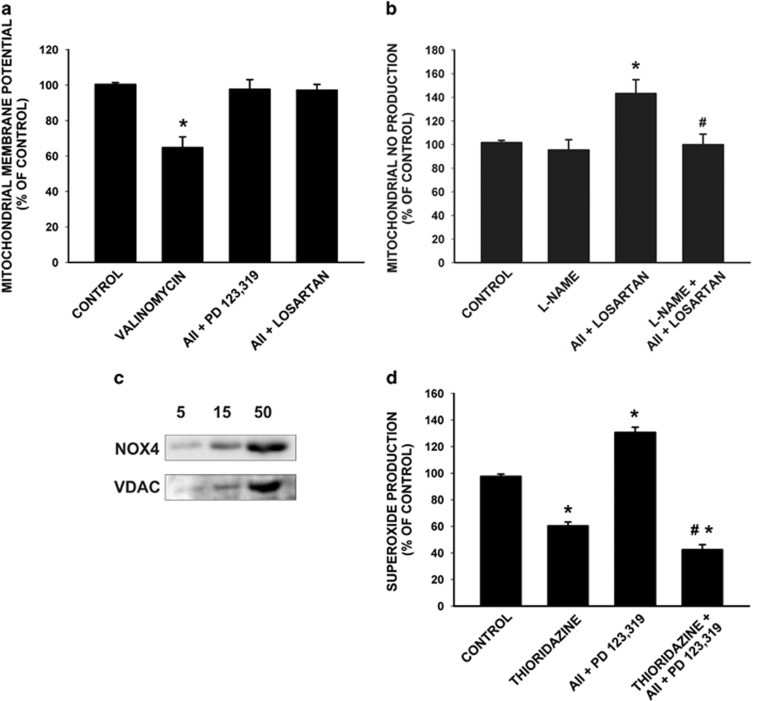
Effect of mitochondrial angiotensin receptors on mitochondrial transmembrane potential, nitric oxide and superoxide production. (**a**) In isolated mitochondria, activation of AT1 and AT2 receptors (AII+ the AT2 blocker PD123,319, and AII+ the AT1 blocker losartan, respectively) did not induce any significant change in mitochondrial membrane potential maintenance; however, the potassium ionophore valinomycin led to loss of approximately 40% of transmembrane potential relative to non-treated mitochondria (*n*=5). (**b**) Activation of AT2 receptors (AII+ the AT1 blocker losartan) induced an increase in levels of NO that was inhibited by pre-treatment with the NOS inhibitor l-NAME (*n*=5–8). (**c**) The presence of Nox4 in isolated mitochondria was also shown by western blot assay with a rabbit monoclonal anti-Nox4 antibody, which detected a band of 60  kDa, and an antibody against the mitochondrial marker VDAC. The Nox4 signal increased with mitochondrial content (5, 10, 50 *μ*g of mitochondrial sample loaded in the acrylamide gel). (**d**) In isolated mitochondria, activation of AT1 receptors with AII (AII+the AT2 receptor antagonist PD123,319) resulted in increased levels of superoxide, and simultaneous treatment with the NOX4 inhibitor thioridazine led to the inhibition of total superoxide to about a 40% of untreated controls (*n*=4–8). Data are mean±S.E.M. *P<0.05 compared with control. ^#^*P*<0.05 compared with the group treated with AII (one-way analysis of variance (ANOVA) and Bonferroni *post hoc* test). l-NAME, l-arginine methyl ester hydrochloride; NOS, nitric oxide synthase

Isolated mitochondria were incubated with the AT1 receptor antagonist losartan or the AT2 receptor antagonist PD123,319, and then incubated with AII. Mitochondria were energized through complex I by adding pyruvate and malate. Activation of AT1 or AT2 receptors did not induce any significant change in mitochondrial membrane potential, indicating that the bioenergetic properties of the mitochondria were not affected (Figure 5a). As control, the potassium ionophore valinomycin was added to the sample at a final concentration of 0.5 *μ*g/ml, which led to loss of approximately 40% of transmembrane potential relative to non-treated mitochondria.

In isolated mitochondria, activation of AT2 receptors (i.e. treatment with AII and the AT1 antagonist losartan) induced an increase in levels of NO that was inhibited by pre-treatment with the NOS inhibitor l-NAME (Figure 5b). This is consistent with the mitochondrial respiration data, which showed that AT2-dependent respiratory inhibition was prevented by treatment of isolated mitochondria with l-NAME, confirming the link between mitochondrial AT2 receptors and NO production, and the mitochondrial respiratory function.

Mitochondrial superoxide (O_2_^−^) production was measured by lucigenin-enhanced chemiluminescence. In isolated mitochondria, activation of AT1 receptors with AII (i.e. treatment with AII and the AT2 receptor antagonist PD123,319) resulted in increased levels of superoxide. The major sources of reactive oxygen species (ROS) in the cell are NADPH oxidase (Nox) proteins and the mitochondrial electron transport chain (ETC). Nox4 is the main intracellular form of the Nox protein family in several types of cells.^16, 17^ In the present study, the presence of Nox4 in rat brain isolated mitochondria was demonstrated using a specific rabbit monoclonal antibody, which showed a 60 kDa band that increased with the amount of mitochondrial sample loaded in the SDS gel (Figure 5c). To clarify the role of Nox4 on superoxide production, we tested the effect of the Nox4 inhibitor thioridazine.^18^ Treatment of isolated mitochondria with AII and the AT2 blocker PD123,319 (i.e. activation of AT1 receptors) and simultaneous treatment with thioridazine led to the inhibition of total superoxide to about a 40% of untreated controls. The remaining superoxide was probably due to mitochondrial electron leakage at the ETC or other sources (Figure 5d). This is consistent with the findings on respiratory function that showed that pre-treatment of isolated mitochondria with the Nox4 inhibitor thioridazine blocked the AT1-induced increase in oxygen consumption (Figures 4a and b). The role of Nox4 in AII-induced superoxide production was confirmed with a second Nox4 inhibitor (diphenyleneidonium, DPI). As observed after treatment with thioridazine (Figure 5d), simultaneous treatment with DPI (AII+PD123,319+DPI) led to inhibition of total superoxide to about 40–50% of untreated controls (data not shown; see Materials and Methods, Superoxide production assay).

## Discussion

Mitochondria play a major role in aging and aging-related neurodegenerative disorders such as degeneration of dopaminergic neurons and PD.^19^ The SN of PD patients shows alteration of mitochondrial NADPH dehydrogenase (complex I) activity, and complex I inhibitors such as MPTP, rotenone and other pesticides cause neurological changes similar to those observed in PD.^20, 21^ Previous immunohistochemical findings from our group suggest the possible existence of an intracellular or intracrine RAS in dopaminergic neurons of some mammalian species, including humans.^5, 13^ The present study demonstrates the presence of AII and the main AII receptors (i.e. AT1 and AT2) in the mitochondria of dopaminergic neurons by immunofluorescence and electron microscopy. This was also observed using fluorescent-tagged angiotensin receptors and fluorescent angiotensin. The results were confirmed in mitochondrial fractions isolated from the nigral region of rat and the dopaminergic neuron cell line MES 23.5. Our results show a differential distribution of both types of AII receptors; while AT2 were mainly cytoplasmic and colocalized with mitochondrial labeling, AT1 had more superficial and nuclear distribution, although we also found AT1 receptors in mitochondria. Furthermore, there was a clear difference in the expression of AT1 and AT2 in the mitochondrial fraction, with AT2 being much more abundant than AT1.

The present findings show that mitochondrial AT1 and AT2 receptor activation produced opposite effects on respiratory rates, which is consistent with that observed in AT1 and AT2 cell surface receptors. Activation of AT2 receptors induced a moderate but significant decrease in active and maximal respiration (OXPHOS and ETS). The effect was confirmed in mitochondria isolated from mice deficient in AT2 receptors, which showed an increase in respiratory rates. The possible mechanisms involved in these effects were then investigated. We found that mitochondrial AT2 receptor activation induced an increase in mitochondrial NO levels, which was blocked by inhibition of mitochondrial NOS activity by l-NAME, which also blocked the reduction in respiratory activity induced by AT2 activation, indicating that NO mediates this process and may act as a respiratory modulator. Initial observations considered NO as proapoptotic; however, it is now known that this occurs only at high concentrations of NO, and that physiological levels of NO are antiapoptotic.^22^ At physiological concentrations, NO competes with oxygen by the active site of cytochrome oxidase. Short- term inhibition of complex IV by low nonlethal levels of NO inhibits respiration and initiates a protective action to maintain the membrane potential, which results in protection of the cell against further damage and prevents apoptosis.^23, 24^ AT2-mediated regulation of mitochondrial respiration may represent an unrecognized primary line of defence against oxidative stress and stress-associated damage. It is known that levels of oxidative stress are increased in dopaminergic neurons, probably related to dopamine metabolism, and the above-mentioned defence mechanism may be particularly important against dopaminergic degeneration and PD.

Mitochondrial AT1 activation produced an increase in oxygen consumption, and increased generation of superoxide via mitochondrial Nox4. This effect was confirmed in mitochondria isolated from mice deficient in AT1 receptors, which showed a decrease in respiratory rates, and is also consistent with previous observations for cell surface AT1 receptors and membrane NADPH oxidase. Our previous studies in animal models of PD showed that AII, via cell surface AT1 receptors, increases oxidative stress through activation of membrane-bound NADPH oxidase.^7, 9, 25^ Within the mitochondria, the primary site of ROS generation is the ETC, as leakage of electrons at complexes I and III leads to a partial reduction of oxygen to form O_2_^−^. In addition, the Nox4 isoform, a member of the NADPH oxidase family, has been localized in intracellular membranes of cardiomyocytes, and renal cells.^16, 26, 27^ In the present study, we demonstrated expression of Nox4 in mitochondria isolated from the nigral region, which is consistent with the results of previous studies that showed the involvement of Nox4 in mitochondrial O_2_^−^ production using small interfering RNA (siRNA) in isolated mitochondria from renal cells.^16^

In addition to the effect of mitochondrial AT1 receptors mentioned above, the activation of cell surface AT1 receptors and membrane-bound NADPH oxidase may also affect mitochondrial function. A number of studies have shown a ROS-mediated interaction (i.e. cross-talk signaling) between the membrane-bound NADPH oxidase complex and mitochondria, so that ROS generated by NADPH oxidase may act as a trigger to induce the opening of ATP-sensitive potassium channels (mitoKATP), which leads to generation of mitochondrial ROS.^28^ In cultures of dopaminergic neurons, we previously observed that inhibition of mitoKATP channels inhibits the AII-induced increase in O_2_^−^ production.^29^ Mitochondrial AT1 and AT2 receptors may play a major role in maintaining the integrity of this essential organelle against extra-mitochondrial insults, at least in the early moments, as observed in the present experiments with isolated mitochondria. Consistent with this, an increase in cell levels of oxidative stress (i.e. treatment of dopaminergic cells with very low doses of MPP^+^) led to an increase in the expression of mitochondrial AT2 receptors. Interestingly, the present findings also show that aging modifies mitochondrial AII receptor expression and the above-mentioned response to the increase in cell levels of oxidative stress. A number of previous studies have shown proinflammatory and pro-oxidative changes in the SN and different tissues of aged rats, and that age-related changes in RAS activity are involved in these changes and in the increased vulnerability of dopaminergic neurons with aging.^30, 31^ Although an upregulation of mitochondrial AT2 receptors in response to the aging-related oxidative state may be expected, the present study shows an increase in mitochondrial AT1 receptor expression and a decrease in mitochondrial AT2 receptor expression in mitochondria isolated from old rats relative to those isolated from young rats. This may contribute to the increased vulnerability of these cells by inducing more mitochondrial oxidative stress and changes in respiratory efficiency, and play an important role in the development of aged-related neurodegenerative disorders such as PD.^32, 33^

**Figure 6:**
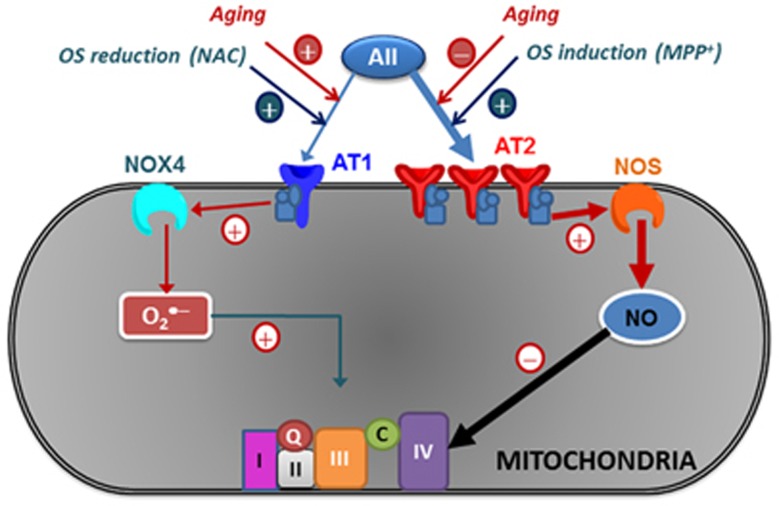
Model of the role that AT1 and AT2 receptors play in modulating oxidative phosphorylation in brain mitochondria. Activation of AT1 receptors in mitochondria regulates superoxide production, via Nox4, and increases respiration. Mitochondrial AT2 receptors are much more abundant and induce, via nitric oxide, a decrease in mitochondrial respiration, modulating oxidative phosphorylation without significant alteration in mitochondrial membrane potential, which indicates that the bioenergetic properties of the mitochondria are not affected. Mitochondrial AT2 receptor expression increased after treatment of cells with oxidative stress (OS) inducers (such as low doses of MPP^+^) and decreased with aging. Mitochondrial AT1 expression increased with aging and after treatment of cells with antioxidants (such as *N*-acetyl-cysteine, NAC). At mitochondrial level, AT2 receptors may act as respiratory modulators and counteract low levels of OS, which may be particularly important in cells with an increase in levels of OS such as dopaminergic neurons. Aging induces altered expression of mitochondrial AT1 and AT2 receptors that may induce mitochondrial dysfunction, the main risk factor for neurodegeneration

In summary (Figure 6), we conclude that functional AT1 and AT2 receptors exist in brain mitochondria, and particularly in dopaminergic neurons. Activation of mitochondrial AT1 induced superoxide production, via mitochondrial NOX4, and increased both mitochondrial respiration and ETS_max_. Mitochondrial AT2 activation caused an NO-mediated reduction of mitochondrial respiration and ETS_max_, modulating oxidative phosphorylation without significant alteration in mitochondrial membrane potential, which indicates that the bioenergetic properties of the mitochondria are not affected. Mitochondrial AT2 receptors, which are clearly more abundant compared with mitochondrial AT1 in young rats, may counteract or modulate at the mitochondrial level the pro-oxidative effects of AII stimulation of the cell membrane and mitochondrial AT1 receptors. AT2-mediated regulation of mitochondrial respiration may represent an unrecognized primary line of defence against oxidative stress, which may be particularly important in neurons with increased levels of oxidative stress such as dopaminergic neurons.

An increase in mitochondrial AT1 and a decrease in mitochondrial AT2 expression in the nigral region of aged rats relative to young rats may play a major role in the mitochondrial dysfunction associated with normal aging, which is the major risk factor for the development of PD and other neurodegenerative diseases. Different types of drugs acting on the local RAS are currently used in vascular and renal diseases, and have been suggested as potential treatments for neurodegenerative diseases, including PD, based on their effects on surface AII receptors. However, the functional effects of mitochondrial receptors, particularly AT2 receptors, must be taken into account in the design of new therapeutic strategies, and especially in diseases associated with excessive oxidative stress such as PD, but also diabetes, obesity and most of the cardiovascular diseases.

## Materials and Methods

### Experimental design

Brain sections from adult male Sprague-Dawley rat containing SN, primary cultures from the nigral region and cultures of the dopaminergic neuron cell line MES 23.5 were used to investigate the presence of AII receptors in mitochondria from dopaminergic neurons by immunofluorescence and electron microscopy. All experiments were carried out in accordance with Directive 2010/63/EU and Directive 86/609/CEE and were approved by the corresponding committee at the University of Santiago de Compostela. Animals were housed at constant room temperature (RT) (21–22° C) and 12-h light/dark cycle. All surgery was performed under ketamine/xylazine anesthesia. The localization of angiotensinogen and angiotensin receptors in dopaminergic neurons isolated from rat SN was confirmed by LCM and RT-PCR. In addition, pure isolated mitochondria from the nigral region of rat were used to confirm the expression of AII receptors in mitochondria using WB assay. Dopaminergic cells were also transfected with fluorescence-tagged AT1 and AT2 receptors, or treated with fluorescent AII to investigate colocalization with mitochondrial markers.

Mitochondria isolated from the nigral region of rat in the VM and from AT1 and AT2 receptor knockout mice (Agtr1a C57BL/6 background and Agtr2 FVB background mutant mice) brain were used to investigate the ability of AII receptors to modulate mitochondrial respiration, mitochondrial membrane potential and mitochondrial superoxide and nitric oxide (NO) production. For this purpose, mitochondria were incubated with different compounds to assess the effects of activation with either AT1 or AT2 on respiratory capacity and maintenance of membrane potential. Mitochondria were treated with AII (1 nM) and pre-incubated with the AT2 antagonist PD123,319 (2 *μ*M) or the AT1 antagonist losartan (3 *μ*M), and/or the NADPH oxidase, NOX4, inhibitor thioridazine (10 *μ*M) or the NOS inhibitor l-NAME (100 *μ*M). The concentrations of the above-mentioned compounds were experimentally determined as the most appropriate for evaluating the studied effects. Mitochondrial oxidative stress was estimated measuring superoxide production (i.e. the most important reactive oxygen species, ROS, in mitochondria) using a sensitive luminescence-based assay. Finally, RT-PCR of nigral dopaminergic neurons isolated by LCM and WB assays of mitochondria isolated from young adult and aged male Sprague-Dawley rats (10 weeks and 20 months respectively) were used to investigate age-related changes in dopaminergic neuron and mitochondrial AII receptor expression.

### Primary cultures from the nigral region and cultures of the MES 23.5 dopaminergic neurons

Ventral mesencephalic tissue was dissected from rat embryos of 14 days of gestation (E14). The tissue was incubated in 0.1% trypsin (Sigma, St. Louis, MO, USA), 0.05% DNase (Sigma) and DMEM (Invitrogen, Paisley, Scotland, UK) for 20 min at 37 °C, and was then washed in DNase/DMEM and mechanically dissociated. The resulting cell suspension was centrifuged at 50 × *g* for 5 min, the supernatant was carefully removed and the pellet resuspended in 0.05% DNase/DMEM to the final volume required. The number of viable cells in the suspension was estimated with acridine orange/ethidium bromide. Cells were plated onto 35-mm culture dishes (Falcon; Becton Dickinson, Franklin Lakes, NJ, USA) previously coated with poly-l-lysine (100 *μ*g/ml; Sigma) and laminin (4 *μ*g/ml; Sigma). The cells were seeded at a density of 1.5 × 10^5^ cells/cm^2^ and maintained under control conditions (DMEM/HAMS F12/(1 : 1) containing 10% fetal bovine serum (FBS; Biochrom KG, Berlin, Germany)). The cell cultures were maintained in a humidified CO_2_ incubator (5% CO_2_; 37 °C) for 8 days *in vitro* (DIV; see below); the entire culture medium was removed on day 2 and replaced with fresh culture medium.

Dopaminergic MES 23.5 cells, a gift from Dr. Wei-Dong Le (Baylor College of Medicine, Houston, TX, USA), were cultured in DMEM/F12 containing Sato's components growth medium supplemented with 2% FBS, 100 units/ml penicillin and 100 *μ*g/ml streptomycin at 37 °C in a humidified CO_2_ incubator (5% CO_2_, 95% air).^34^ MES 23.5 cells were plated at a density of 0.5 × 10^5^/cm^2^ onto 35-mm plastic dishes with glass coverslips previously coated with poly-l-lysine (Sigma; 10 mg/ml). To enhance differentiation, cells were stimulated by adding dibutyryl-cAMP (D0627, Sigma; 1 mM) to the supplemented growth medium.

### Triple fluorescent labeling of cultures

Cultures grown on glass coverslips were incubated with the fluorescent probe Mitotracker Deep Red (MTDR; 20 nM; Molecular Probes, Waltham, MA, USA) for mitochondrial labeling. After mitochondrial labeling, cultures were fixed with 4% paraformaldehyde in Dulbecco's phosphate-buffered saline (pH 7.4) for 20 min, and were then processed for double immunofluorescence fluorescence. Cultures were incubated at 4 °C with a mouse monoclonal anti-TH (Sigma; 1 : 30 000) antibody as dopaminergic marker and goat polyclonal anti-AT1 (sc-31181; 1 : 50; Santa Cruz Biotechnology) or rabbit polyclonal anti-AT2 receptor (sc-9040; 1 : 50; Santa Cruz Biotechnology) antibodies. The immunoreaction was visualized with the following fluorescent secondary antibodies: Alexa Fluor 405-conjugated donkey anti-mouse IgG (1 : 200; Abcam, Cambridge, England, UK) or biotinylated horse anti-goat IgG (BA9500, 1 : 50; Vector Labs, Burlingame, CA, USA) or biotinylated swine anti-rabbit IgG (1 : 200; Dako, Barcelona, Spain) plus Alexa Fluor 488-conjugated Streptavidin (1 : 2500; Molecular Probes). Colocalization of markers was confirmed by confocal laser microscopy (TCS-SP2; Leica, Heidelberg, Germany) performing sequential scan to avoid any potential overlap. In all experiments, the control cultures, in which the primary antibody was omitted, were immunonegative for these markers.

### Immuno electron microscopy for TH and AT1 and AT2 receptors

Adult male Sprague-Dawley rats were deeply anesthetized with a mixture of ketamine/xylazine and perfused with saline (0.09% NaCl) followed by a solution of 4% paraformaldehyde and 0.1% glutaraldehyde in 0.1 M phosphate buffer pH 7.4 (PB). After perfusion, brains were quickly removed from the skull, immersed in the same fixative solution overnight at 4 °C and rinsed thoroughly in PB. Free-floating 40 μm coronal sections were obtained on a vibratome and stored in PB until use. Sections containing the SN were transferred to citrate buffer pH 6.0 (Vector Laboratories; H-3300) at RT for 5 min and then to citrate buffer at 80 °C for 30 min. Sections were rinsed consecutively in citrate buffer and phosphate-buffered saline (PBS) pH 7.4 at RT, prior to pre-treatment with 1% sodium borohydride in PBS for 15 min. The sections were then rinsed several times in PBS, and transferred to a solution of 5% hydrogen peroxide in PBS for 30 min, and rinsed several times in PBS. Sections were pre-incubated in 10% normal serum containing 0.01% triton X-100 in PBS for 1 h and then incubated for 72 h at 4 °C with the appropriate primary antibody: mouse monoclonal anti-TH (Sigma; 1 : 10 000), and AT1 goat polyclonal diluted 1 : 100 or AT2 rabbit polyclonal diluted 1 : 200, Santa Cruz Biotechnologies catalog number sc-31181 and sc-9040, respectively. Sections were rinsed thoroughly in PBS and incubated for 1 h with the appropriate secondary antibodies (biotinylated goat anti-rabbit or horse anti-goat; Vector Laboratories) diluted 1 : 400 in PBS containing 0.01% triton X-100, rinsed in PBS and incubated with an avidin-biotin-complex kit (Vector Laboratories; PK6100, diluted 1 : 100) for 1 h at RT. Sections were rinsed multiple times in PBS, developed using 3,3'-diaminobenzidine peroxidase kit (Vector SK4100) and rinsed in PBS.

Sections immunolabeled for AT1, AT2 or TH were rinsed in PB, transferred to a solution of 1% osmium tetroxide in PB for 1 h at RT and rinsed in PB prior to gradual dehydration in 50–70% ethanol. The sections were then transferred to a contrast solution containing 1% uranyl acetate in 70% ethanol for 1 h at RT. Sections were then rinsed in 70% ethanol to remove excess uranyl acetate and gradually dehydrated in 5 min baths of 70–100% ethanol. Sections were cleared in propylene oxide and gradually infiltrated with Epon resin by sequential immersion in a 2 : 1 mixture of propylene oxide and Epon resin (30 min), 1 : 1 mixture of propylene oxide and Epon resin (1 h), 1 : 2 mixture of propylene oxide and epon (1 h) and finally transferring the sections to Epon resin overnight at 4 °C. The following day the sections were transferred to freshly prepared Epon resin for 1 h at RT, flat embedded and allowed to polymerize for a minimum of 72 h at 60 °C. After flat embedding was completed, the SN was clearly identified using a brightfield microscope and re-dissected for ultramicrotomy. Semithin (1-*μ*m-thick) and ultrathin (90-nm-thick) sections were cut using a Leica EM UC6 ultramicrotome (Leica Microsystems; Wetzlar, Germany). Ultrathin sections were placed on copper grids and observed and photographed using a Hitachi transmission electron microscopy (Hitachi, Tokyo, Japan) equipped with a Hamamatsu Orca digital camera (Hamamatsu, Hamamatsu, Japan).

### Specificity of antibodies

The specificity of the antibodies used for WB and immunolabeling studies was established in previous studies: AT1 sc-31181^35^ and AT2 sc-9040.^36, 37^ In addition, the specificity of the antibodies was confirmed in our laboratory by preadsortion with the corresponding synthetic peptide antigen.^38^ In the present study, we also used WB analysis of lysates from HEK293 cells transfected with AT1 or AT2 tagged to fusion tail DDK (TA50011 from Origene, Rockville, MD, USA; DDK tag: DYKDDDDK). The specificity of the antibodies was confirmed by the presence of a predominant immunoreactive band in positively transfected lysates and the absence of this band in negative controls, which consisted of lysates transfected with empty vectors (Figure 1b).

### Retrograde tracing, LCM and RT-PCR

Young adult (10-week-old, *n*=3) and aged (18–20-month-old, *n*=4) male Sprague-Dawley rats were used for LCM studies. Rats received three intrastriatal injections of red retrobeads (0,5 *μ*l/injection; Lumafluor Inc., Durham, NC, USA) under ketamine/medetomidine anesthesia. The tracer was injected using a 10 *μ*l Hamilton syringe at a rate of 0.1 *μ*l/min. The stereotaxic coordinates for the injections were: bregma: +1, −0.1 or −1.2 mm; lateral: ±3, ±3.7 or ±4.5 mm; ventral: 5 mm from dura; tooth bar: 0. Forty-eight hours after injection, rats were killed, brains were removed, snap-frozen in liquid nitrogen and stored at −80 °C. Serial coronal sections (20-*μ*m-thick) including the VM were cut on a cryotome and mounted on RNase-free ultraviolet light-treated glass slides. Slides were immediately frozen at −80 °C, and stored in sterile Falcon tubes containing silica gel. A series of SN sections were processed for immunofluorescence with an antibody against TH in order to verify the localization of red retrobeads within nigral dopaminergic neurons. LCM was performed using a PALM laser microbeam system (Zeiss, Jena, Germany) with a fluorescence option. Before LCM, frozen sections were allowed to equilibrate to increasing temperatures (−20 °C, 4 °C, RT), fixed with ethanol (70, 96, 100, 100%) and dried at RT. Red retrobeads-labeled neurons were visualized (× 40 objective) within the SN, marked under fluorescence illumination and then cut and catapulted under brightfield microscopy. Neuronal cell pools were catapulted into an adhesive cap (Zeiss). For RNA extraction, trizol (Invitrogen) was added directly through the lid, the tube was shaken upside-down and then spun. Two thousand labeled dopaminergic neurons per animal were used for total RNA extraction according to the manufacturer's instructions. Homogenates of rat VM were used as a positive control.

Total RNA was reverse-transcribed to cDNA with nucleoside triphosphates containing deoxyribose, random primers and M-MLV reverse transcriptase (50U; Invitrogen). Real-time PCR was used to examine the relative levels of TH, angiotensinogen, AT1 and AT2 mRNA. A real-time iCycler PCR platform (Bio Rad, Hercules, CA, USA) and IQ SYBR Green Supermix kit (Bio Rad) were used. *β*-Actin was used as a housekeeping gene and the data were evaluated by the delta–delta Ct method (2−ΔΔCt), where Ct is the cycle threshold. Finally, the PCR products were loaded on a 2% agarose gel with SYBR Safe stain (Invitrogen) and separated by electrophoresis. Primer sequences were as follows: for AT1, forward 5′-TTCAACCTCTACGCCAGTGTG-3′, reverse 5′-GCCAAGCCAGCCATCAGC-3′ for AT2, forward 5′-AACATCTGCTGAAGACCAATAG-3′, reverse 5′-AGAAGGTCAGAACATGGAAGG-3′ for angiotensinogen, forward 5′-GAGTGAGGCAAGAGGTGTA-3′, reverse 5′-TCCAACGATCCAAGGTAGAA-3′ for TH, forward 5′-GGCTTCTCTGACCAGGTGTATCG-3′, reverse 5′-GCAATCTCTTCCGCTGTGTATTCC-3′ and for *β*-actin, forward 5′-TCGTGCGTGACATTAAAGAG-3′, reverse 5′-TGCCACAGGATTCCATACC-3′.

### Transient transfection of AII receptors and Alexa Fluor 488-conjugated AII treatment of dopaminergic cell line MES 23.5

The dopaminergic neuron cell line MES 23.5 was cultured in DMEM/F12 containing Sato's components growth medium supplemented with 2% FBS, 100 units/ml penicillin and 100 *μ*g/ml streptomycin at 37 °C in a humidified CO_2_ incubator (5% CO_2_, 95% air). MES 23.5 cells were plated at a density of 0.3 × 10^5^/cm^2^ onto 35-mm plastic dishes with glass coverslips previously coated with poly-l-lysine (Sigma; 10 mg/ml). In the first series of experiments, cells were treated with MitoTracker Deep Red 633 (20 nM, 15 min) to label mitochondria, and transiently transfected with 1 *μ*g of AT1 cDNA (AT1/EGFP-N3) and 3 μg of AT2 cDNA (AT2/YFP-N1) for 24 h using Lipofectamine LTX transfection reagent (Invitrogen). To study the effects of pro-oxidative and antioxidative conditions, some cultures of cells transfected with AT2-yellow fluorescent protein (YFP) receptor were treated with a low dose of the dopaminergic neurotoxin MPP^+^ iodide (10 *μ*M; Sigma) for 24 h (*n*=4), and cells transfected with AT1-enhanced green fluorescent protein (EGFP) receptor were treated with the antioxidant *N*-acetyl-l-cysteine (0.5 mM; Sigma) for 24 h (*n*=3). The effect of these treatments on expression of mitochondrial AT1 and AT2 receptors were observed by confocal microscopy and confirmed by WB of isolated mitochondria from MES 23.5 cells using antibodies against GFP (Life Technologies, Waltham, MA, USA; G10362; 1 : 800), AT1 and AT2 (Santa Cruz Biotechnology; sc-31181 and sc-9040) and VDAC/porin (Sigma; V2139; 1 : 1,000). The purity of isolated mitochondria from MES 23.5 cells was previously demonstrated by the absence of other cellular compartment markers (see below). In order to minimize the toxicity of AT1 receptor overexpression, cells transfected with AT1-EGFP required the simultaneous treatment with the AT1 receptor antagonist losartan (3 *μ*M), which was essential to maintain cell survival.

In a second series of experiments we investigated the presence of AII in mitochondria. MES 23.5 cells were treated with the mitochondrial marker MitoTracker Deep Red 633 (20 nM, 15 min) and Alexa Fluor 488-conjugated AII (500 nM; Molecular Probes). Cells were fixed in 4% paraformaldehyde for different lengths of time between 30 min and 8 h, and fluorescence images were acquired with a Leica SP5 confocal laser scanning microscope.

### Mitochondrial isolation

Mitochondria from nigral region and whole brain of rat, and from AT1a and AT2 receptor knockout mice (Agtr1a and Agtr2 mutant mice) brain, were isolated and purified according to the protocol described by Sims and Anderson^14^ with some modifications. This protocol combines differential centrifugation and discontinuous Percoll density gradient centrifugation to isolate pure mitochondria with scarce contamination by synaptosomes and myelin. Rat nigral region was removed and washed in cold isolation buffer (0.32 M sucrose, 1 mM and 10 mM TRIS; pH 7.4). The tissue was cut into small pieces, transferred to a Dounce homogenizer with 12% Percoll solution, and then homogenized on ice using a loose fitting and tight fitting glass pestles. The homogenate was slowly layered on a previously prepared discontinuous Percoll gradient consisting of 26% Percoll layered over 40% Percoll, and centrifuged using a fixed-angle rotor at 30 700 × *g* for 5 min at 4 °C. This produced three separate bands, and the enriched mitochondrial fraction, which appeared at the interface between the 26 and 40% Percoll layers, was carefully removed with a glass Pasteur pipette. The mitochondrial fraction was diluted by adding isolation buffer, and was again centrifuged at 16 700 × *g* for 10 min at 4 °C. This yielded a mitochondrial pellet, which was gently resuspended in the residual supernatant. Finally, the pellet was resuspended in isolation buffer and centrifuged at 7300 × *g* for 10 min at 4 °C, yielding a pellet of pure mitochondria that was used for WB and functional assays. For WB confirmation of the presence of transfected angiotensin receptors in mitochondria of the dopaminergic cell line MES 23.5, mitochondria were isolated with a Mitochondria Isolation kit (Mitosciences; ab110170) for cultured cells.

### Western blot of mitochondria from rat nigral region and MES 23.5 dopaminergic neurons

Protein concentration in the isolated mitochondria from the nigral region (*n*=4) and whole homogenate (*n*=4) were quantified using the Pierce BCA Protein Assay Kit (Thermo Scientific, Fremont, CA, USA). Equal amounts of protein were separated on a 5–10% bis-tris polyacrylamide gel, and transferred to nitrocellulose membranes. Membranes were incubated overnight with primary antibodies against VDAC/porin (V2139, 1 : 1000) and *α*-Tubulin (T5168, 1 : 50 000) from Sigma, HDAC2 (sc-56685, 1 : 200), AT1 (sc-31181, 1 : 200) and AT2 (sc-9040, 1 : 200) from Santa Cruz Biotechnology and rabbit monoclonal antibody to Nox4 (ab133303, 1 : 800) from Abcam. The following HRP-conjugated secondary antibodies were used: Protein A (1 : 5000) (NA9120V; GE Healthcare), chicken anti-mouse IgG-HRP (sc-2954, 1 : 2500) and donkey anti-goat IgG-HRP (sc-2020, 1 : 2500) from Santa Cruz Biotechnology. Immunoreactive bands were detected with an Immun-Star HRP Chemiluminescent Kit (170-5044; Bio Rad) and visualized with a chemiluminescence detection system (Molecular Imager ChemiDoc XRS System; Bio Rad). Specificity of the antibodies was confirmed as indicated above.

### Detection of AII in purified isolated mitochondria by HPLC and specific AII EIA

For the separation of angiotensin peptides, mitochondria were isolated as described above and stored at −20 °C. The mitochondrial pellet was reconstituted in 100 *μ*l of Milli-Q water and heated at 96 °C for 15 min. The mitochondrial fraction was acidified with heptafluorobutyric acid (HFBA) to a final concentration of 0.1%, sonicated and centrifuged at 20 000 × *g* for 20 min at 4 °C. The supernatants were applied to a Sep Pak columns (Oasis HLB 1cc, WAT094225) pre-conditioned with 1 ml of methanol and deionized water. The loaded Sep Pak columns were washed with 1 ml of 0.1% HFBA in deionized water. AII and its fragments were eluted with 1 ml of methanol in 0.1% of HFBA, and the eluent dried in a vacuum concentrator (Savant ISS110). Dried samples (*n*=5) were resuspended in 60 *μ*l of a solution of 17% acetonitrile in 4 mM TEAF with 30 mM formic acid and injected (20 *μ*l/injection) into the HPLC system. Peptides were separated at 35 °C and a flow rate of 1 ml/min with an acetonitrile gradient on a reverse phase C18 column (Waters Symmetry300C18; 150 × 3.9 mm, 5 *μ*m particle size; Waters, Barcelona, Spain). For the acetonitrile gradient, solution A was made with 30 mM formic acid in 4 mM TEAF. Solution B consisted of 90% acetonitrile in 4 mM TEAF with 30 mM formic acid. The linear gradient used was from 11 to 50% of B in 20 min. Eluate from the column was monitored at a wavelength of 220 nm in an UV-Vis detector (SPD-20AV; Shimadzu, Columbia, MD, USA). AII fractions were collected with a fraction collector (FRC-10A; Shimadzu), dried in a vacuum concentrator and stored at −80 °C until analysis of AII content by EIA kit (A05880; SpiBio, Frankston, TX, USA) following the manufacturer's specifications.

### High-resolution respirometry

Mitochondrial respiration was measured by high-resolution respirometry at 37 °C using an Oxygraph-2k respirometer (OROBOROS Instruments, Innsbruck, Austria). Aliquots of 80–120 *μ*g of isolated mitochondria were incubated with AT1 or AT2 receptor antagonists (3 *μ*M losartan or 2 *μ*M PD123,319, respectively; *n*=3–8) for 3 min, and treated with 1 nM AII for 5 min. To know the effects of NO and NOX4, the NOS inhibitor l-NAME or the NOX4 inhibitor thioridazine were added to mitochondria (see below) with or without corresponding treatment in respiration buffer (125 mM KCl, 5 mM HEPES, 3 mM MgCl_2_, 2 mM KH2PO_4_ and 0.5 mM EGTA; pH 7.4) using a substrate-uncoupler-inhibitor titration protocol (SUIT), as previously described.^39^ The substrates used to fuel the ETS were 2 mM malate+5 mM pyruvate (MP) for evaluating complex I respiration. The OXPHOS capacity, which is similar to State 3, was measured in the presence of substrates with saturating ADP (2.5 mM). The maximum ETS capacity (ETS_max_; i.e. non-coupled respiration) was evaluated using carbonyl cyanide-4-(trifluoromethoxy)phenylhydrazone (FCCP; 0.5–1.5 *μ*M) as the uncoupler. The integrity and function of our mitochondrial preparations were confirmed by addition of exogenous cytochrome *c*, which did not enhance mitochondrial respiration, indicating nonsignificant damage to the outer mitochondrial membrane during isolation procedures (data not shown). Respiration rates (O_2_ flux) were calculated as the negative time derivative of oxygen concentration, and Datlab software (OROBOROS Instruments) was used for both data acquisition and analysis. Respiration rates were normalized to the amount of mitochondrial protein to allow comparison between groups without the influence of differences in the amount of mitochondria loaded into the chamber.

### Mitochondrial membrane potential

Membrane potential of mitochondria isolated from rat brain was measured using the sensitive lipophilic cationic fluorescent probe 5,5′,6,6′-tetrachoro-1,1′,3,3′-tetraethylbenzimidazolyl-carbocyanine iodide (JC-1; Sigma) as previously described.^40^ Isolated mitochondria were treated with different compounds and respiratory substrates for 10 min at 37 °C. Mitochondria were treated with AII (1 nM) and the AT2 antagonist PD123,319 (2 *μ*M) or the AT1 antagonist losartan (3 *μ*M) to investigate the effect of mitochondrial AT1 and AT2 receptors on mitochondrial membrane potential. Samples (*n*=5) were then stained with JC-1 (0.2 *μ*g/ml; previously incubated on ice for 20 min) and the red fluorescence of dye agglomerates was measured for 15 min (excitation/emission wavelength=490/590 nm) in a fluorescence plate reader (Tecan; Infinite M200). Valinomycin (0.5 *μ*g/ml), a potassium ionophore, was used as a control to permeabilize the mitochondrial membrane for K^+^ ions, dissipating the mitochondrial electrochemical potential and preventing JC-1 aggregation.

### Superoxide production and nitric oxide production assays

Pure mitochondrial fractions obtained with Percoll gradients were directly assayed for superoxide (O_2_^−^) production using lucigenin-enhanced chemiluminescence as follows.^16, 17^ Lucigenin is an acridylium dinitrate compound that on reducing and interacting with superoxide anions emits light that is quantified to measure the production of O_2_^−^. An aliquot of 30 *μ*g mitochondria (*n*=4–8) was incubated for 10 min at 37 °C with 1 nM AII and pre-incubated with the AT2 receptor antagonist PD123,319 (2 *μ*M) in 50 mM phosphate buffer, pH 7.0 containing 1 mM EGTA and 150 mM sucrose. Five micromolar lucigenin and 100 *μ*M NADPH were then added to start the reaction. Photon emission, in terms of relative chemiluminescence units (RLU), was measured every 20 s for 10 min using a luminometer plate reader (Tecan; Infinite M200; Männedorf, Switzerland). There was no measurable activity in the absence of NADPH. The Nox4 inhibitor thioridazine (10 *μ*M) was used to determine the amount of O_2_^−^ produced by this mitochondrial member of the Nox/NADPH oxidase family. The role of Nox4 in AII-induced superoxide production was confirmed with a second Nox4 inhibitor (diphenyleneidonium, DPI). However, it has been suggested that DPI may also affect NOS.^41, 42^ Mitochondrial NOS is related to the mitochondrial AT2 receptor effects and inhibition with DPI may be not adequate for the present study, particularly for respirometry. Therefore, we only included the results observed with thioridazine.

Mitochondrial nitric oxide (NO) production was measured with an NO fluorometric assay kit (Biovision, Milpitas, CA, USA). Because of its short half-life, direct measurement of NO is difficult; thus the total concentrations of nitrate and nitrite have been used as useful indicators of NO production. An aliquot of 30 *μ*g of mitochondria (*n*=5–8) was incubated for 10 min with 1 nM AII and the AT1 antagonist losartan (3 *μ*M) to investigate the effect of mitochondrial AT2 receptors activation, and the effect of the NOS inhibitor l-NAME (100 *μ*M). Nitrate was then converted to nitrite by the nitrate reductase enzyme, and the total nitrite concentration was measured as nitrite reacted with the fluorescent probe DAN (2,3-diaminonaphthalene), which is proportional to the total nitric oxide production.

### LDH and COX activities

LDH is a cytosolic enzyme predominantly associated with contaminating synaptosomes; thus, it was used to assess the purity of the mitochondrial fraction. LDH activity was measured in 2.5 *μ*g isolated mitochondria and whole homogenate protein (*n*=6), according to the manufacturer's specifications (Roche 04744926001). The micromol/min/mg was calculated using LDH enzyme (Sigma, L-3916) as a reference standard.

The COX activity was used to normalize the data obtained from AT1 and AT2 receptor expression performed by WB in aged rats, as this provides the most accurate estimation of mitochondrial content. COX activity was measured in isolated mitochondria using a commercial kit according to the manufacturer's specifications (KC310100; Biochain, Newark, CA, USA). COX activity was measured in a 96-well plate by detecting the decrease in absorbance of ferrocytochrome *c* at 550 nm, as it is oxidized to ferricytochrome *c* by COX. Ten micrograms of isolated mitochondria protein with 0.1% of DTT were assayed adding ferrocytochrome *c* substrate and reading the absorption at 550 nm immediately for 55 s. The COX activity (unit/ml) of each sample was calculated using the molar absorbance coefficient 21.84 for ferrocytochrome *c* and ferricytochrome *c* at 550 nm

### Statistical analysis

All data were obtained from at least three independent experiments and were expressed as mean values±S.E.M. Two-group comparisons were analyzed by a Student's *t*-test and multiple comparisons were analyzed by one-way ANOVA followed by a *post hoc* Bonferroni test. The normality of populations and homogeneity of variances were tested before each ANOVA. Differences were considered significant at *P*<0.05. Statistical analyses were carried out with SigmaStat 3.0 (Jandel Scientific, San Rafael, CA, USA).

## Abbreviations

AII: angiotensin II
AT1: angiotensin type 1 receptors
AT2: angiotensin type 2 receptors
BCA: bicinchoninic acid
COX: cytochrome *c* oxidase
DPI: diphenyleneidonium
E: maximum respiratory rate
ETC: electron transport chain
ETS: electron transport system
HDAC2: histone deacetylase 2
LCM: laser captured microdissection
LDH: lactate dehydrogenase
mitoKATP: ATP-sensitive potassium channels
MTDR: Mitotracker Deep Red
NAC: *N*-acetyl-cysteine
NO: nitric oxide
NOS: nitric oxide synthase
Nox4: NADPH oxidase 4
O_2_^−^: superoxide
P: oxidative phosphorylation
PD: Parkinson's disease
RAS: renin–angiotensin system
ROS: reactive oxygen species
RT-PCR: reverse trascription polymerase chain reaction
SN: substantia nigra
SNc: substantia nigra pars compacta
TH: tyrosine hydroxylase
VDAC: voltage-dependent anion channel
VM: ventral mesencephalon
WB: western blot
